# The study of radiosensitivity in left handed compared to right handed healthy women

**DOI:** 10.1186/1756-6649-12-3

**Published:** 2012-08-24

**Authors:** Meysam Khosravifarsani, Ali Shabestani Monfared, Haleh Akhavan-Niaki, Dariush Moslemi, Karimollah Hajian-Tilaki, Farideh Elahimanesh, Sajad Borzoueisileh, Nayer Seyfizadeh, Mehrangiz Amiri

**Affiliations:** 1 Radiobiology and Radiation Protection, Cellular & Molecular Biology Research Center, Babol University of Medical Sciences, Babol, Iran; 2 Medical Physics, Cellular & Molecular Biology Research Center, Babol University of Medical Sciences, Babol, Iran; 3 Cellular and Molecular Biology, Cellular & Molecular Biology Research Center, Babol University of Medical Sciences, Babol, Iran; 4 Radiation Oncology, Shahid Rajae Hospital, Babolsar, Iran; 5 Statistic and Epidemiology, Babol University of Medical Sciences, Babol, Iran; 6 Clinical Biochemistry, Babol University of Medical Sciences, Babol, Iran; 7 Nuclear Medicine, Babol University of Medical Sciences, Babol, Iran

## Abstract

**Background:**

Radiosensitivity is an inheriting trait that mainly depends on genetic factors. it is well known in similar dose of ionizing radiation and identical biological characteristics 9–10 percent of normal population have higher radiation response. Some reports indicate that distribution of breast cancer, immune diseases including autoimmune diseases as example lupus, Myasthenia Gravies and even the rate of allergy are more frequent in left handed individuals compared to right handed individuals. The main goal of the present study is determination of radiosensitivity in left handed compared to right handed in healthy women by cytokinesis blocked micronuclei [CBMN] assay.

5 ml peripheral fresh blood sample was taken from 100 healthy women including 60 right handed and 40 left handed. The age of participants was between 20–25 old years and they had been matched by sex. After blood sampling, blood samples were divided to 2 groups including irradiated and non-irradiated lymphocytes that irradiated lymphocytes were exposed to 2 Gy CO-60 Gama rays source then chromosomal aberrations was analyzed by CBMN [Cytokinesis Blocked Micronuclei Assay].

**Results:**

Our results have shown radiosensitivity index [RI] in left-handers compared to right-handers is higher. Furthermore, the mean MN frequency is elevated in irradiated lymphocytes of left-handers in comparison with right-handers.

**Conclusion:**

Our results from CBMN assay have shown radiosensitivity in the left handed is higher than right handed women but more attempts need to prove this hypothesis.

## Background

The Main goal of radiosensitivity recognition is radiation protection of radiation workers, identification of cancer patients which are radiosensitive to ionizing radiation after treating by radiotherapy and other treatment methods by ionizing radiation, identification of outcomes from atomic disasters and individualization of radiosensitivity in astronauts which are exposed to cosmic rays [1]. In clinical radiotherapy, the patients that receive similar physical dose of ionizing radiation have different response to radiotherapy from latent to sever and sometimes lethal. Clinical radiotherapy of cancer patients have shown that 5–7 percent of cancer patients have adverse side effects in their normal tissues after clinical radiotherapy. These side effects are including late side effects, early side effects and cancer induction [2]. Some reports have released that radiosensitivity is an inheriting trait in which genetic factors have a main role [3]. The association of some SNPs and radiosensitivity has been described in several genes [1,4-7]. Previous studies demonstrated that genes such as ATM and NBS which are involved in DNA repair mechanisms and cell cycle check point are responsible for hypersensitivity [8]. Other studies described an association between genes such as LRRTM1 [9] and PCSK6 [10] and left handedness. It is well known that except genetic factors other parameters such as physical dose specifications or environmental conditions are important in radiosensitivity of cell and tissue. In similar specifications of physical dose and same environmental conditions it has been reported that some individuals have higher radiation response to ionizing radiations [2]. Investigations on breast cancer distribution among postmenopausal women have also shown that left handed patients are approximately twice of right handed ones [11]. Left handedness has been associated with some disorders such as thyroid and immune disorder especially autoimmune disease like lupus, multiple sclerosis, allergy, and migraine [12]. All of these findings lead us to the study of radiosensitivity between the left- and right handed women by CBMN assay.

## Materials and methods

### Blood sampling

Before bloods sampling all of participants filled patient consent form then 5 ml peripheral fresh blood sample was taken from 100 healthy women including 60 right handed and 40 left handed. Blood samples were then divided into two identical parts which one part was used as control-vehicle and the second part considered as exposed-vehicle. Environmental conditions for control and exposed vehicles were identical and blood sample transferring was close to ice pack. The age of participants was between 20–25 years and all of them were female. We used questionnaire about handedness of participants and their family. Handedness of participants was determined by oral questioning. History of cancer, previous irradiation, smoking, drug treatment and alcohol usage in participants and their family were considered as exclusion criteria.

### Irradiation

Irradiation of peripheral blood samples was by 2 Gy of CO-60 gamma rays source (Theratone780 manufactured by Canada) and the dose rate of 120 CGy/min. Source to samples distance (SSD) was 80 cm and exposure area was 10 × 10 cm^2^. After irradiation, control and exposed blood samples were transferred for cell culture and were incubated in 37°C for 72 hr.

### CBMN (Cytokinesis Blocked Micronuclei Assay)

CBMN assay was performed by IAEA protocol [13]. 0.5 ml peripheral blood sample was added to 4.5 ml RPMI-1640 medium supplemented by 10% fetal calf serum, 1% Glutamine, 1% Penicillin/Streptomycin then 100 microliter Phytohaemagglutinin (PHA-M) was added to the medium. After 44 hr 100 microliter Cytochalasin B (6 μgr/ml diluted in 200 ml DMSO and 19.8 ml DW) was added to the medium. Cytochalasin B is an inhibitor of cytokinesis which prohibit from cell division in cytokinesis stage of cell cycle. 72 hr After PHA (SIGMA) addition, blood samples were harvested and then centrifuged with 2000 RPM for 10 min (BOECHO U-320 R). After which supernatant was wasted and 2–3 ml fresh hypotonic solution 0.075 M KCL was added and centrifuged with 1200 RPM for 7 min again then supernatant was wasted and 5 ml fixture solution containing methanol and acetic acid (6/1 methanol: glacial acetic acid) was added quickly. After 20 min the tubes were centrifuged (1200 RPM for 7 min) then supernatant was wasted. Fixation was repeated three times with similar procedure for good separation of binuclear lymphocytes. Afterward binuclear lymphocytes present in the pellet were dropped on cleaned and cooled slides. After 24 hr slides were stained by Giemsa (Diluted in PBS) 5% for 20 min then slides were washed by deionized water and air dried. All the slides were observed by light microscope in 40× magnification using SAIRAN microscope. All of the slides were coded before analyzing. Micronuclei were scored in 1000 binucleated (BN) cells and scoring was double blind. The micronuclei were scored according to the scoring criteria proposed by Fenech [14,15]. The ratio of mean frequency of MN in exposed to non-exposed samples in each group was considered as radiosensitivity index [16].

### Statistical analyze

Statistical analyze was performed by SPSS-16 software. Paired sample t-test was made between control and exposed group and independent sample t-test carried out between right handed and left handed subjects. P-Value ≤ 0.05 was considered as significance level.

## Results

**Figure 1:**
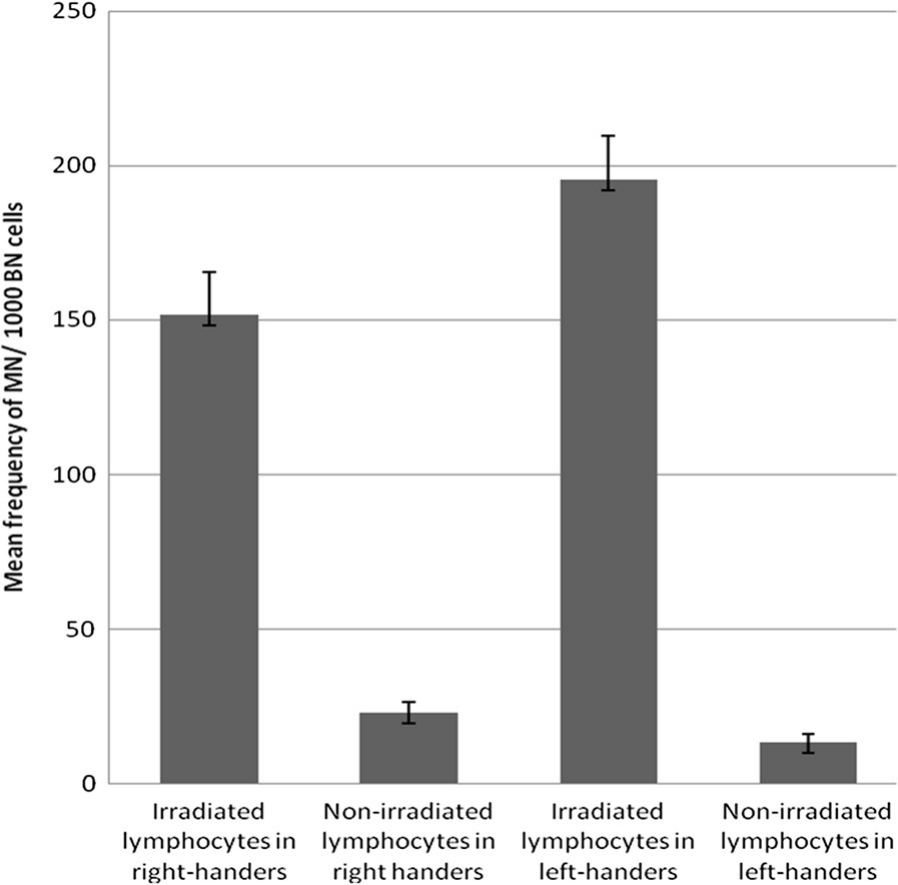
Mean frequency of MN in right handed and left handed women.

As shown by our results, mean frequency of MN for exposed compared to control group was significantly higher in both left handed and right handed subjects (P = .000) (Figure 1).

Radiosensitivity index for left-handers was 14.8(195.4/13.2), whereas for right-handers was 6.59(151.65/23). Mean frequency of MN in non-irradiated lymphocytes of right-handers was higher than left-handers and this difference was statistically significant (P = 0.001) but mean frequency of MN for irradiated lymphocytes of right-handers was lower than left-handers and difference is statistically significant (P = 0.000) (Table 1).

**Table 1:** Mean frequency of MN/ 1000 BN cells in right-handers and left-handers

		**N**	**Mean±SD**
Irradiated_lymphocytes	Right-handers	60	151.65±13.89
	Left-handers	40	195.40±14.14
Non_irradiated lymphocytes	Right-handers	60	23.00±3.46
	Left-handers	40	13.20±2.67

We performed data analysis between irradiated and non_irradiated lymphocytes separately; our results released that mean frequency of MN observed in non-irradiated lymphocytes from right handed group compared to non-irradiated lymphocytes from left handed group is remarkably higher (P = 0.001). Furthermore the mean MN frequency observed in irradiated lymphocytes from right handed group compared to irradiated lymphocytes from left handed group is significantly elevated (P = 0.000).

## Discussion and conclusion

Our results have shown that mean frequency of MN in irradiated lymphocytes is higher than non_irradiated lymphocytes in both right handed and left handed group. These findings are similar to former studies performed by Silva et al. [17], Koksal G et al. [18] and Gantonberg HW et al. [19]. Our results have released that radiosensitivity index is elevated in left handed women compared to right handed women and also it's evident that difference of the mean MN frequency in left-handers compared to right-handers is higher. These findings are in agreement with another study reported by khosravifarsani et al. [16]. Geschwind et al. have observed higher frequencies of immune disorders especially bowel, gut, learning disability, migraine and myasthenia gravis in left-handers and their relatives compared to right-handers [12].

The study of Made K Ramadhani et al. have shown that the distribution of breast cancer in postmenopausal women is more frequent in left handed compared to right handed women [11]. Also Fritsch et al. showed that the risk of developing post-menopausal breast cancer in women is significantly higher in left-handers compared to right-handers [20]. Stellman et al. showed a high association of left handedness with disease and disease risk factors [21]. The study of Ashton [22] Coren and Halpern [23], Fleminger et al. [24], Kuhlemier [25], Lalumiere et al. [26], Lansky [27]; Porac et al. [28] and Tan, have revealed shortened life span in left handed individuals compared to right handed individuals [29]. Investigations of Binali et al displayed that caries experience in left handed individuals have lower incidence than right handed individuals [30]. S Geschwind and Galaburda have declared high proportion of mental retardation and developmental problems in left handed individuals [31]. Former study demonstrated that the ABO blood groups are in relation with the radiation response in carcinoma of the cervix. On the basis of this study, O blood group has poorer response to radiotherapy than A and B merged and B blood group has lower response to ionizing radiation than A blood group [32].

Molecular and genome wide association studies are consistent with a polygenic model of handedness. Few genes have been suggested as candidates for the establishment of hand preference such as the imprinted gene LRRTM1 on 2p12-q11 [9]*,* PCSK6 gene on 15q26.3 [10] and the X-linked androgen receptor located on Xq11-12 [33,34].

Although among few SNPs detected in left handed subjects none was studied as a risk factor for radiosensitivity, but in light of our findings one may hypothesis that those SNPs may either directly modify radiosensitivity by influencing DNA repair pathways or indirectly as part of a haplotype harboring a polymorphism involved in cell cycle control or DNA repair. In fact a recent molecular study indicates that carriers of MSH2 gIVS12-6nt-C allele show an increased radiosensitivity [35]. MSH2 is one of the genes involved in DNA mismatch repair and located on chromosome 2 at 2p21. It has also been shown that polymorphism in Rad51 a gene located on chromosome 15 at 15q15.1 and involved in double strand break repair, can enhance cancer risk and in vitro chromosomal radiosensitivity [4,5]. It has also been postulated that genetic polymorphism of androgen receptor may act as a risk-modifier for BRCA2-associated breast cancers [36].

Although our results have shown the association of left handedness and radiosensitivity, but to our knowledge no SNPs linked to DNA repair mechanisms or cell cycle check points have been studied in this regard. Further molecular investigations are needed to understand the possible genetic basis of this association.

## Competing interest

The authors declare that they have no conflict of interest.

## Authors’ contributions

MKH: wrote article, participated in the design of study and carried out the CBMN assay ASHM: conceived the study, irradiation of blood samples. KHT: performed the statistical analysis FE, SB, NS and MA: participated in CBMN assay. HAN and DM: participated in the design of the study, revision of manuscript. All authors read and approved the final manuscript.

## Pre-publication history

The pre-publication history for this paper can be accessed here:

http://www.biomedcentral.com/1756-6649/12/3/prepub
